# A novel strategy for the MPPT in a photovoltaic system via sliding modes control

**DOI:** 10.1371/journal.pone.0311831

**Published:** 2024-12-13

**Authors:** Itzel Contreras Carmona, Belem Saldivar, Otniel Portillo-Rodríguez, Víctor Manuel Ramírez Rivera, Leopoldo Gil Antonio, Juan Manuel Jacinto-Villegas

**Affiliations:** 1 Facultad de Ingeniería, Universidad Autónoma del Estado de México, Toluca, México; 2 Departamento de Control Automático, Centro de Investigación y de Estudios Avanzados del Instituto Politécnico Nacional, México City, México; 3 Departamento de Energía Renovable, Centro de Investigación Científica de Yucatán (CICY), Yucatán, México; 4 Investigadoras e Investigadores por México CONAHCYT, Ciudad de México, México; 5 TecNM: Tecnológico de Estudios Superiores de Jocotitlán, Ejido de San Juan y San Agustín Jocotitlán, México; SR University, INDIA

## Abstract

This paper proposes a robust maximum power point tracking algorithm based on a super twisting sliding modes controller. The underlying idea is solving the classical trajectory tracking control problem where the maximum power point defines the reference path. This trajectory is determined through two approaches: a) using the simplest linear and multiple regression models that can be constructed from the solar irradiance and temperature, and b) considering optimum operating parameters derived from the photovoltaic system’s characteristics. The proposal is compared with the classical methods Perturbation and Observation and Incremental Conductance, as well as with two recently reported hybrid algorithm based on Artificial Neural Networks: one uses the Levenberg-Marquardt algorithm and the other applies Bayesian regularization to generate current and voltage references, respectively. Both use a Proportional-Integral-Derivative controller to solve the maximum power point tracking problem. Numerical simulations confirm the effectiveness of the method proposed in this work regarding convergence time, power efficiency, and amplitude of oscillations. Furthermore, it has been shown that, although no significant differences in the system response are observed with respect to the Artificial Neural Networks-based methods, the proposed algorithm with a reference generated through a linear regression constitutes a low-complexity solution that does not require a temperature sensor to efficiently solve the maximum power point tracking problem.

## Introduction

There is an increasing demand for new supplies of clean, renewable energy that reduce the use of resources harmful to the environment. Photovoltaic (PV) systems are a primary renewable energy source [1]. The electricity production through PV energy and its efficient use contribute to sustainable development.

One of the main drawbacks of using PV systems is that the energy conversion efficiency tends to be low; therefore, solar energy is not fully harnessed [2]. PV systems have variable operating points over time and depend on external factors such as irradiance and temperature. These factors decrease the PV system performance by affecting mainly the output power, the fill factor, and the efficiency of the module [3].

In addition, PV systems installed in areas with obstacles that prevent proper reception of solar irradiance tend to reduce electrical power generation. Therefore, it is necessary to use a robust Maximum Power Point (MPP) tracker to obtain the maximum power of the photovoltaic panels, considering variations in temperature and solar irradiance.

One of the classical methods to track the MPP is the Perturbation and Observation (P&O) algorithm. Among its disadvantages we can mention that it provides an oscillatory response around the MPP and does not work correctly in the event of sudden changes in irradiance or temperature. Another classical method for the Maximum Power Point Tracking (MPPT) is the Incremental Conductance (INC) algorithm, which has the disadvantage of slow convergence to the MPP when the increment size is small. Additionally, it can be unstable due to the use of the derivative operation, and the results can be unsatisfactory at low levels of solar irradiance [4].

The different algorithms proposed in the literature to solve the MPPT problem can be classified as described below.

- **Mathematical model-based algorithms**: They use mathematical models to predict the duty cycle (D) value at which a DC-DC converter extracts the maximum power from the PV as a function of solar irradiance and temperature. Among them, we can mention the Fractional Short Circuit Current (FSCC) [5], Fractional Open Circuit Voltage (FOCV) [6], and Extremum Seeking Control (ESC)-based algorithms [7, 8].
- **Sampled data-based algorithms**: These algorithms do not use a PV model, they are easy to implement in practice, and are, therefore, the most widely used. Their operation is based on on the continuous measurement of the PV current, voltage, and power to predict the D value at which a DC-DC converter extracts the maximum power. The most popular methods based on sampled data are P&O [9, 10], INC [11], and Parasitic Capacitance (PC) [12].
- **Traditional regression algorithms**: These algorithms use a regression model to approximate the relation between power output and solar panel input variables to predict the D value at which a DC-DC converter will extract the maximum power. Regression techniques include linear regression, polynomial regression, multiple regression, and non-parametric regression [13, 14].
- **Metaheuristic optimization algorithms**: In these methods, the output power of a PV system is represented as a cost function whose maximum value is defined by the MPP. Then, the determination of the D value at which a DC-DC converter extracts the maximum power is seen as an optimization problem. These algorithms seek to improve MPPT efficiency by exploring different possibilities in the search space. Examples of this type of algorithms are Particle Swarm Optimization (PSO) [15–17], Ant Colony Optimization (ACO) [18], Firefly Algorithm (FFA) [19], Simulated Annealing (SA) [20], Artificial Hummingbird Algorithm [21], Golden Eagle Optimization (GEO) [22], Arithmetic Optimization Algorithm [23], and Runge-Kutta Optimization [24], Marine Predator Algorithm [25].
- **Intelligent Algorithms**: These algorithms use Artificial Intelligence (AI) to determine the MPP; among them, we can mention Artificial Neural Networks (ANN)-based methods [26–31], Support Vector Machines (SVM) [32], Regression Trees (RT) [33], K-Nearest Neighbor Regression (KNR) [34] and Gaussian Process Regression (GPR) [35].
- **Hybrid algorithms**: The hybrid approach combines the strengths of multiple MPPT algorithms to mitigate the limitations of individual algorithms and provide a more robust and adaptive solution for varying environmental conditions [36]. The hybrid algorithms setup incorporates control strategies (Proportional-Integral (PI) controller [23, 32], Proportional-Integral-Derivative (PID) controller [27, 28, 35], differential flatness-based controller [17], nonlinear backstepping terminal sliding mode controller [31], nonlinear generalized global sliding mode controller [30], and fuzzy logic-based controller [37, 38]), with intelligent and/or metaheuristic optimization MPPT algorithms. The combined MPPT algorithms employ a two-step tracking methodology. The first step entails estimating the MPP to define a set-point, and the second step consists in determining the actual MPP using linear or nonlinear controllers [36, 39–41].

This article proposes a new hybrid algorithm for solving the MPPT problem using a Super Twisting Sliding Modes Controller (ST-SMC). The key idea is to solve the trajectory tracking problem of a predefined reference that, in this case, corresponds to the voltage or current at the MPP. This reference signal is generated through two different approaches:

1. By using multiple regressions with irradiance and temperature as inputs, and also considering a linear regression with irradiance as a single input.
2. By using a characterization of the PV system optimal operating parameters, considering the relations given in [42, 43].

In order to assess the performance of the proposed hybrid algorithm in the presence of sudden variations of temperature and irradiance, comparisons are made with two classical methods (P&O [9] and INC [11]) and with two hybrid algorithms (ANN-PID). These hybrid algorithms were recently reported in the literature, and both use an ANN to generate voltage [27] or current [28] references, and both propose the use of a PID controller to solve the MPPT problem.

The structure of the article is organized as follows. After the Introduction, the modeling of the PV system is presented. Then, ST-SMC that solves the trajectory tracking problem is described. Next, for the generation of reference signals to track the MPP, multiple and linear regressions are derived. As explained in what follows, alternatively, the references can be characterized in terms of the optimal operating parameters of the panel. Then, numerical simulation results are presented to illustrate the performance of the proposed hybrid approach, considering abrupt variations of temperature and irradiance. A comparative analysis with the P&O and INC as well as with two ANN-PID algorithms is also presented. Finally, a discussion and some concluding remarks are provided.

## Modeling of the PV system

**Fig 1:**
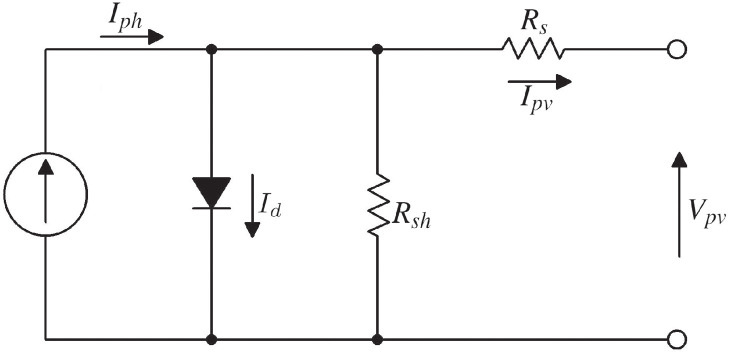
Equivalent circuit of a PV cell.

A PV cell generates electricity through the photoelectric effect. PV cells are connected in series and parallel configurations to constitute a PV module and achieve efficient power output. Depending on its complexity, a PV cell can be represented by several equivalent circuit models, including the single-diode model, commonly used for its simplicity and reasonable accuracy. The equivalent circuit of a PV cell is shown in Fig 1.

The circuit consists of a current source (*I*_*ph*_) which depends on the solar irradiance (*I*_*r*_), a diode with a reverse saturation current (*I*_*d*_), a parallel resistor (*R*_*sh*_) expressing a leakage current, and a series resistor (*R*_*s*_) describing an internal resistance to current flow [44].

The PV module’s output current *I*_*pv*_ can be described by Eq (1), where it is assumed that *R*_*s*_ < < *R*_*sh*_ [45],

$$\begin{array}{c} I_{pv}=N_{p}I_{ph}-N_{p}I_{o}[\text{exp}(\frac{q(\frac{V_{pv}}{N_{s}}+\frac{R_{s}I_{pv}}{N_{p}})}{KT_{c}A})-1] \end{array}$$ (1)

where:

*I*_*pv*_ is the output current of the module

*I*_*ph*_ is the photo-generated current of each cell

*V*_*pv*_ is the output voltage of the module

*N*_*s*_ is the number of cells connected in series

*N*_*p*_ is the number of cells connected in parallel

*q* is the electron charge (1.602 × 10^−19^*C*)

*K* is the Boltzmann’s constant $\left(1.381\times 10^{-23}\frac{J}{K}\right)$

*T*_*c*_ is the temperature of the module in Celsius degrees

*I*_*o*_ is the reverse saturation current of the diode

*A* is the diode ideality factor

Substituting in Eq (1) the parameters of a specific PV module, it is possible to generate its *I*_*pv*_-*V*_*pv*_ and *P*_*pv*_-*V*_*pv*_ curves (for more details see [46]). From these curves, it is possible to determine the MPP, i.e., the values of *V*_*pv*_ or *I*_*pv*_ that must be present at the PV module terminals so that maximum power is transferred to the load.

If the magnitude of *V*_*pv*_ is modulated, the magnitude of *I*_*pv*_ is adjusted according to Eq (1) and vice versa, that is, if magnitude of *I*_*pv*_ is modulated, the magnitude of *V*_*pv*_ is automatically adjusted. To modulate the signals *V*_*pv*_ or *I*_*pv*_, a controllable and variable impedance between the PV module and the load is needed. Buck and Boost DC-DC converters have been the most widely used circuits for this purpose. The former reduces the voltage and increases the current delivered to the load; the latter increases the voltage and reduces the current supplied to the load. Both DC-DC converters have a high efficiency so that most of the power extracted from the PV module can be delivered to the load [47]. The Boost converter has power efficiencies ranging from 90% to 100% [48]. In PV systems, the Boost converter is used to increase the voltage from the solar panel to a level suitable for charging batteries or powering electrical devices, for example.

**Fig 2:**
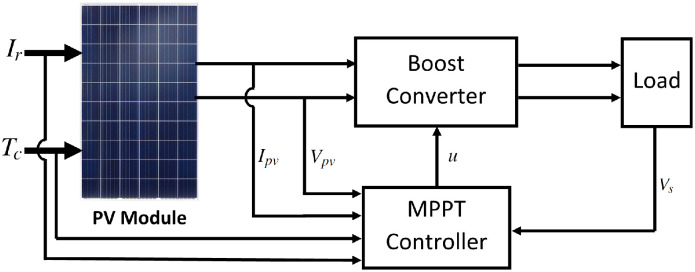
PV system under an MPPT controller.

In this paper, a Boost CD-CD converter is used to numerically determine the MPP of a Renesola JC250^®^ PV module (its parameters are listed in Table 1). The Boost converter is connected to a resistive load, as shown in Fig 2.

**Table 1:** Specifications of the Renesola JC250^®^ PV module.

Parameter	Value
*I*_*r*_	1000 *W*/*m*^2^
*I*_*o*_	1.9164 × 10^−9^ A
*R*_*s*_	0.29838 Ω
*N*_*s*_	60 cells
*I*_*m*_	8.21 A
*V*_*m*_	30.1 V

**Fig 3:**
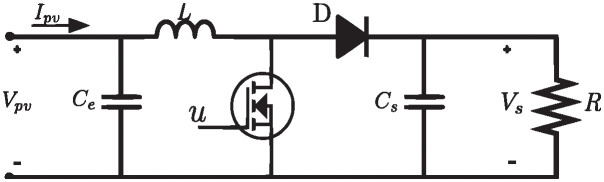
Boost converter topology.

The topology of the circuit is shown in Fig 3, where *V*_*pv*_ is the input voltage and *V*_*s*_ is the output voltage. The inductor current is assumed to be equal to the PV system current *I*_*pv*_.

The components *R*, *L*, *C*_*e*_, *C*_*s*_, are the converter load, the inductance, the input capacitance, and the output capacitance, respectively. The dynamics of the converter is described by a model that combines the equations resulting from the activation and deactivation of the switch *u* (control input). All components are assumed to be ideal (e.g., *u* has zero resistance when closed and infinite resistance when open; therefore, it does not dissipate power).

The model is described by [49]:

$$\begin{array}{c} \frac{dI_{pv}}{dt}=\frac{V_{pv}}{L}-\frac{\left(1-u\right)V_{s}}{L} \end{array}$$ (2)

$$\begin{array}{c} \frac{dV_{s}}{dt}=\frac{\left(1-u\right)I_{pv}}{C_{s}}-\frac{V_{s}}{RC_{s}} \end{array}$$ (3)

The model (2) and (3) can be represented as:

$$\begin{array}{ccc} \dot{x} & = & f(x)+g(x)u\\ y & = & Cx \end{array}$$

where

*x* = [*x*_1_*x*_2_]^*T*^ = [*I*_*pv*_*V*_*s*_]^*T*^, *u* ∈ [0, 1], and *C* = [01].

## Super twisting sliding mode control

The ST algorithm is a second-order SMC applicable to systems with relative degree one, derived from the variable structure theory. The advantage of this algorithm is that it does not require the time-derivative of the sliding variables and significantly reduces the chattering phenomena in the output signal [50].

Its ease of implementation, robustness, and good dynamic response are other significant advantages of the ST-SMC. This technique has been successfully applied to DC-DC converters, see for instance [51].

It is important to point out that there are no major assumptions to be taken into account in applying this control strategy. Here, it is assumed here that the model accurately describes the system and that the dynamics are known. However, the ST-SMC can operate satisfactorily even in the presence of uncertainties and disturbances, if the controller gains are properly tuned. In practice, it must be kept in mind that high frequency switching signals are involved and a sufficiently fast sampling rate is required. Therefore, the selected hardware must be able to operate considering these features.

Two steps are required for the development of the ST-SMC. The first step consists in designing a sliding surface *s* such that when *s* = 0, the desired behavior of the DC-DC converter is achieved. The second step is to design a control law that allows guaranteeing *s* = 0, indefinitely.

The ST-SMC requires the formulation of a switching controller *u*_*st*_ along with an equivalent controller *u*_*eq*_, i.e.,

$$\begin{array}{c} u=u_{st}+u_{eq} \end{array}$$ (4)

The switching strategy is responsible for driving, through the available control action, the state of the system towards the sliding surface. Once the state trajectory reaches the sliding surface, the equivalent controller is responsible for keeping it evolving indefinitely on this surface.

In our case, the control objective is to steer *I*_*pv*_ to a current reference signal *I*_*MPP*_, corresponding to the current at the MPP. A sliding surface can be defined as in [52] to deal with the MPPT problem:

$$\begin{array}{c} s_{1}=I_{pv}-I_{MPP} \end{array}$$ (5)

In an alternative scenario, the sliding surface can be designed to make the output voltage *V*_*pv*_ reach a reference voltage *V*_*MPP*_, corresponding to the voltage at the MPP. In this case, the sliding surface takes the form:

$$\begin{array}{c} s_{2}=V_{pv}-V_{MPP} \end{array}$$ (6)

The equivalent control *u*_*eq*_ is proposed as in [53] as:

$$\begin{array}{c} u_{eq}=1-\frac{V_{pv}}{V_{s}} \end{array}$$ (7)

where *V*_*s*_ is the system output voltage. The switching control *u*_*st*_ is given by [54]:

$$\begin{array}{c} u_{st}=-\lambda {\left|s_{i}\right|}^{\frac{1}{2}}sign\left(s_{i}\right)-\Upsilon \int_{0}^{t}sign\left(s_{i}\left(d\tau \right)\right)d\tau \end{array}$$ (8)

where *s*_*i*_, *i* ∈ {1, 2}, and λ, *Υ* are design parameters. This specific controller structure is known as ST-SMC.

A stability analysis of the proposed ST-SMC that solves the trajectory tracking problem is presented in the S1 Appendix.

In the following sections, two different approaches to generate the reference signals required by the controller to achieve MPPT are presented.

## Multiple and linear regressions

Linear and multiple regressions allow the identification of the relation between one or several independent variables, respectively, to one dependent variable by fitting a linear model to the available data. In our case, the independent variables correspond to the irradiance *I*_*r*_ and temperature *T*_*c*_ while the dependent variables are *I*_*MPP*_ and *V*_*MPP*_.

For the Renesola JC250^®^ PV module, the following regression models were fitted:

1. Two models (planes) to determine *I*_*MPP*_ and *V*_*MPP*_ from the panel’s irradiance and temperature using a multiple regression (MR),
2. A model (straight line) to determine *I*_*MPP*_ from the panel’s irradiance using a linear regression (LR).

The characterization of the reference models to generate *I*_*MPP*_ and *V*_*MPP*_ is obtained from a dataset of 63000 *I*_*pv*_-*V*_*pv*_ and *P*_*pv*_-*V*_*pv*_ curves. These curves were generated by considering variations of the irradiance *I*_*r*_ from 100 *W*/*m*^2^ to 1150 *W*/*m*^2^, with increments of 1 *W*/*m*^2^. For each value of *I*_*r*_, the temperature *T*_*c*_ was varied from 5°C to 65°C, with increments of 1°C. For each pair (*I*_*r*_, *T*_*c*_), *I*_*pv*_ values were determined through the variation of *V*_*pv*_ from 0 V to 38 V, with increments of 0.1 V, by considering the relation (1).

In what follows, the subscripts *LR* and *MR* indicate that the references were obtained through linear and multiple regressions, respectively.

**Fig 4:**
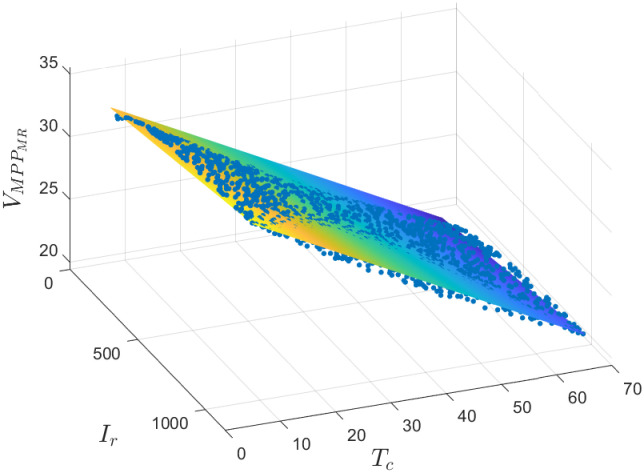
Regression model to predict *V*_*MPP*_ from *I*_*r*_ and *T*_*c*_ using a MR.

The model for predicting *V*_*MPP*_ from *I*_*r*_ and *T*_*c*_ is defined by Eq (9). In Fig 4, one can observe that the data fits into a plane very well (*R*^2^ = 0.9912, *RMSE* = 0.3878); 1500 points with coordinates (*I*_*r*_, *T*_*c*_, $V_{MPP_{MR}}$) randomly sampled from the entire data set are shown.

$$\begin{array}{c} V_{MPP_{MR}}=a_{0}+a_{1}I_{r}+a_{2}T_{c} \end{array}$$ (9)

the values for the coefficients (with 95% confidence bounds) are:

*a*_0_ = 33.9 (33.85, 33.96)
*a*_1_ = 0.002319 (0.002256, 0.002382)
*a*_2_ = −0.2195 (−0.2206, −0.2184)

**Fig 5:**
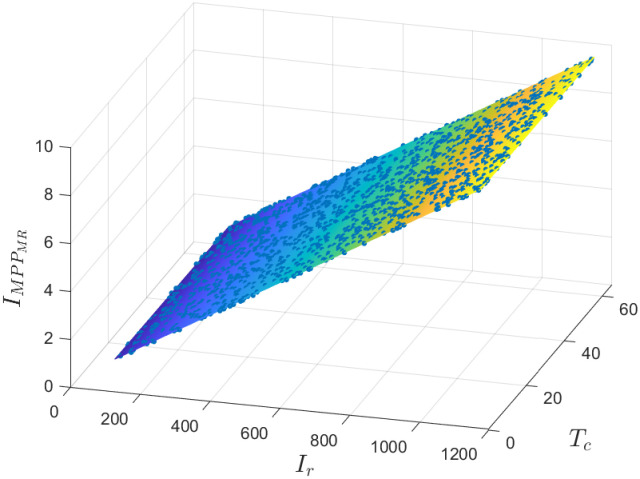
Regression model to predict *I*_*MPP*_ from *I*_*r*_ and *T*_*c*_ using a MR.

The regression model that predicts *I*_*MPP*_ from *I*_*r*_ and *T*_*c*_ is defined by Eq (9). As shown in Fig 5, the data fits into a plane almost perfectly (*R*^2^ = 1.0, *RMSE* = 0.0116); 1500 points with coordinates (*I*_*r*_, *T*_*c*_, $I_{MPP_{MR}}$) randomly sampled from the entire data set are illustrated.

$$\begin{array}{c} I_{MPP_{MR}}=a_{0}+a_{1}I_{r}+a_{2}T_{c} \end{array}$$ (10)

the following values of the coefficients (95% confidence bounds) were obtained:

*a*_0_ = 0.0332(0.03142, 0.03498)
*a*_1_ = 0.008241(0.008239, 0.008243
*a*_2_ = −0.001048(−0.001081, −0.001014)

Once the multiple regression models were exploited to characterize *V*_*MPP*_ and *I*_*MPP*_, we wanted to explore if a prediction of *I*_*MPP*_ can be generated from a linear regression (straight line), that is, if it is possible to characterize *I*_*MPP*_ in terms of a single variable (*T*_*c*_ or *I*_*r*_).

**Fig 6:**
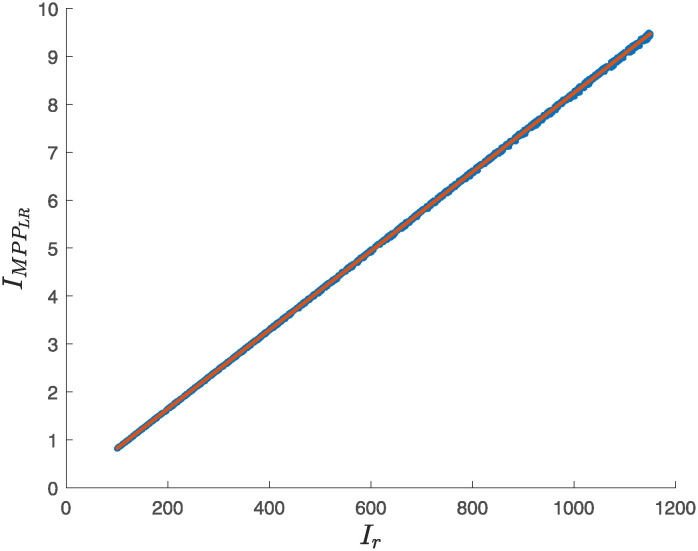
Regression line to predict *I*_*MPP*_ from *I*_*r*_ using a LR.

Analyzing the dataset, we found no correlation between *I*_*MPP*_ and *T*_*c*_ (Pearson’s correlation coefficient *ρ* = −0.0074). However, there is a perfect correlation between *I*_*MPP*_ and *I*_*r*_ (*ρ* = 1.0), i.e., *I*_*MPP*_ can be characterized in terms of *I*_*r*_ through the model described by Eq (11). Fig 6 shows how data fits into a straight line (*R*^2^ = 0.9999, *RMSE* = 0.0218); 1500 points with coordinates (*I*_*r*_, $I_{MPP_{LR}}$) randomly sampled from the entire data set are shown.

$$\begin{array}{c} I_{MPP_{LR}}=a_{0}+a_{1}I_{r} \end{array}$$ (11)

the following values for the coefficients (95% confidence bounds) were obtained:

*a*_0_ = −0.002116(−0.004697, −0.0004656)
*a*_1_ = 0.008239(0.0082395, 0.0082420)

For the case of the *V*_*MPP*_ reference, we found a weak correlation between *V*_*MPP*_ and *I*_*r*_ (*ρ* = 0.1875), and a strong negative correlation between *V*_*MPP*_ and *T*_*c*_ (*ρ* = −0.9787). A regression was performed to determine *V*_*MPP*_ as a function of *T*_*c*_, the resulting model performed very poorly (*R* = 0.9579, *RMSE* = 0.8133), so a linear model will not be used to generate the *V*_*MPP*_ reference.

Note that the *RMSE* of $V_{MPP_{MR}}$ model (0.3878) is higher than the RMSE of $I_{MPP_{MR}}$ (0.0116) and $I_{MPP_{LR}}$ (0.0218) models. Therefore, $I_{MPP_{MR}}$ and $I_{MPP_{LR}}$ models are expected to generate better references to track the MPPT than the $V_{MPP_{MR}}$ model. Because of its simplicity and similar RMSE value, $I_{MPP_{LR}}$ model was chosen over $I_{MPP_{MR}}$.

The reference signals *V*_*MPP*_ and *I*_*MPP*_ that will be considered for the numerical simulations are the ones derived from the relation (9) for the reference voltage, and from (11) for the reference current.

The following section presents an alternative approach to generate the reference signals to track the MPP.

## Panel’s optimum operating parameters

This section explains how the reference signals *I*_*MPP*_ and *V*_*MPP*_ can be characterized in terms of four optimum operating parameters of the panel:

- panel’s short-circuit current *I*_*sc*_,
- panel’s open circuit voltage *V*_*oc*_,
- panel’s output current *I*_*m*_,
- panel’s output voltage *V*_*m*_.

The values of these parameters are determined under optimum or standard conditions, i.e., with an irradiance of 1000 *W*/*m*^2^ and a temperature of 25°C [42, 43].

First, as explained below, the reference current *I*_*MPP*_ is characterized.

The relation between *I*_*pv*_ and *V*_*pv*_ can be characterized by Eq (1).

By considering *N*_*p*_ = 1 and *R*_*s*_ = 0 [55], Eq (1) simplifies to:

$$\begin{array}{c} I_{pv}=I_{ph}-I_{o}\left[exp(BV_{pv})-1\right] \end{array}$$ (12)

$$\begin{array}{c} I_{pv}≅I_{ph}-I_{o}exp(BV_{pv}) \end{array}$$ (13)

where:

$$\begin{array}{c} B=\frac{q}{N_{s}KT_{c}A} \end{array}$$ (14)

If the output terminals of the panel are short-circuited, i.e., *V*_*pv*_ = 0, then *I*_*pv*_ = *I*_*sc*_. Substituting this condition in Eq (12), we obtain:

$$\begin{array}{c} I_{sc}=I_{ph} \end{array}$$ (15)

Now, if the output terminals of the panel are in open circuit condition, i.e., *V*_*pv*_ = *V*_*oc*_, then *I*_*pv*_ = 0. Substituting this condition and Eq (15) in Eq (13), we obtain:

$$\begin{array}{c} I_{o}=I_{sc}exp\left(-BV_{oc}\right) \end{array}$$ (16)

The optimal operating current and voltage parameters, denoted by *I*_*m*_ and *V*_*m*_, respectively, which generate the maximum power at the panel’s standard conditions, are related to *I*_*sc*_ and *V*_*oc*_ through Eq (17), which is derived from Eqs (12)–(16) [55].

$$\begin{array}{c} I_{m}=I_{sc}[1-exp(B(V_{m}-V_{oc}))] \end{array}$$ (17)

Since the values of the parameters *I*_*sc*_, *V*_*oc*_, *I*_*m*_, and *V*_*m*_ can be found in the data-sheet provided by the manufacturer, from Eq (17), the value of *B* can be determined as:

$$\begin{array}{c} B=\frac{1}{V_{m}-V_{oc}}\text{ln}(1-\frac{I_{m}}{I_{sc}}) \end{array}$$ (18)

To properly characterize the MPP at the panel’s nonstandard conditions, additional parameters ($I_{sc}^{\prime }$, $V_{oc}^{\prime }$, $I_{m}^{\prime }$, $V_{m}^{\prime }$, *B*′) must be considered. According to [56], these parameters are related with *I*_*r*_ and *T*_*c*_, as shown in the following equations:

$$\begin{array}{c} I_{sc}^{\prime }=I_{sc}+\frac{I_{r}}{I_{r_{ref}}}\left(1+a\Delta T_{c}\right) \end{array}$$ (19)

$$\begin{array}{c} V_{oc}^{\prime }=V_{oc}\left(1-c\Delta T_{c}\right)\text{ln}\left(e+b\Delta I_{r}\right)) \end{array}$$ (20)

$$\begin{array}{c} I_{m}^{\prime }=I_{m}\frac{I_{r}}{I_{r_{ref}}}\left(1+a\Delta T_{c}\right) \end{array}$$ (21)

$$\begin{array}{c} V_{m}^{\prime }=V_{m}\left(1-c\Delta T_{c}\right)\text{ln}\left(e+b\Delta I_{r}\right) \end{array}$$ (22)

$$\begin{array}{c} B^{\prime }=\frac{V_{oc}^{\prime }}{V_{m}^{\prime }-V_{oc}^{\prime }}\text{ln}(1-\frac{I_{m}^{\prime }}{I_{sc}^{\prime }}) \end{array}$$ (23)

where $I_{r_{ref}}$ and $T_{c_{ref}}$ are solar irradiance and temperature under the standard conditions, and $\Delta T_{c}=T_{c}-T_{c_{ref}}$, $\Delta I_{r}=\frac{I_{r}}{I_{r_{ref}}}-1$.

The typical values of *a*, *b*, *c* are [42]:

*a* = 0.0025/°C

*b* = 0.5 *m*^2^/*W*

*c* = 0.00288/°C

As explained in [42], using the *LambertW*-function, it is possible to define the panel’s current at the MPP based on its optimum operating parameters as:

$$\begin{array}{c} I_{MPP_{OC}}=I_{sc}^{\prime }[1-\frac{1}{LambertW(exp^{\left(B^{\prime }+1\right)})}] \end{array}$$ (24)

Similarly, the panel’s voltage at the MPP can be characterized through Eq (25) [43]:

$$\begin{array}{c} V_{MPP_{OC}}=\frac{1}{B^{\prime }}[LambertW(e^{B^{\prime }V_{oc}^{\prime }+1}+e)-1] \end{array}$$ (25)

The subscript *OC* in (24) and (25) indicates that the reference signal (current or voltage) was derived by using the panel’s operating parameters at optimum conditions. These reference signals are considered for the ST-SMC to track the MPP, as will be explained in the next section.

## Numerical simulations results

This section presents numerical simulations results obtained by considering the PV system in closed loop with the ST-SMC algorithm.

For the case of the sliding surface *s*_1_ defined in (5) (in terms of current), the reference signals that will be considered are:

1. Reference current derived through the linear regression technique $I_{MPP_{LR}}$ defined in (11),
2. Reference current derived through the panel’s optimal operating parameters $I_{MPP_{OC}}$ defined in (24).

For the sliding surface *s*_2_ stated in (6) (in terms of voltage) the reference signals are:

1. Reference voltage derived through the multiple regression technique $V_{MPP_{MR}}$ defined in (9),
2. Reference voltage derived through the panel’s optimal operating parameters $V_{MPP_{OC}}$ defined in (25).

Table 2 shows the numerical values of the Boost converter parameters. These values were calculated based on the voltage and current delivered by the PV module and the load applied to the system; in this case, a resistive load is taken.

**Table 2:** Boost converter parameters.

Parameter	Value
Load resistance	12 Ω
Output capacitance	470 *μf*
Operation frequency PWM	45 Khz
Inductance	10 mH
Input capacitance	330 *μf*

The gains λ, Υ of the switching control *u*_*st*_ defined in Eq (8) are chosen as in [57]: λ = 0.1, Υ = 0.01.

To challenge the performance of the ST-SMC strategy, abrupt variations of external conditions (temperature and irradiance) are considered. The three different scenarios described below were considered.

**Fig 7:**
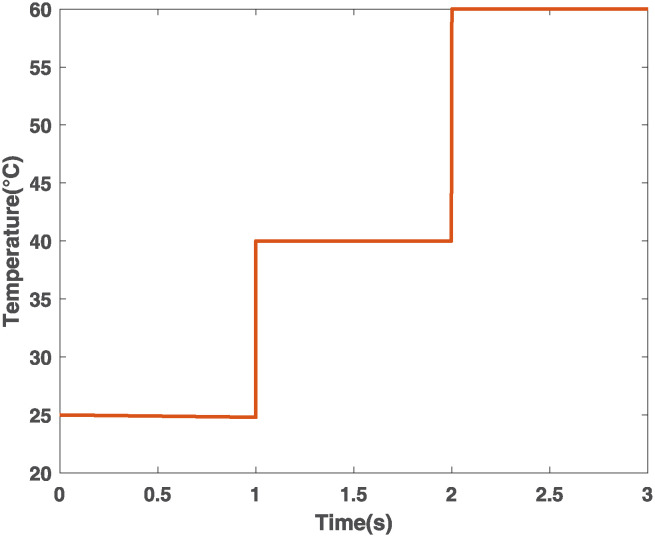
Case 1. Temperature variations.

**Case 1 (Temperature variations).** From 0 to 1 s, the temperature is 25°C; from 1 to 2 s, it increases from 25°C to 40°C; and from 2 to 3 s, an increment from 40°C to 60°C is considered. Solar irradiance remains constant at 1000 $\frac{W}{m^{2}}$. This scenario is shown in Fig 7.

**Fig 8:**
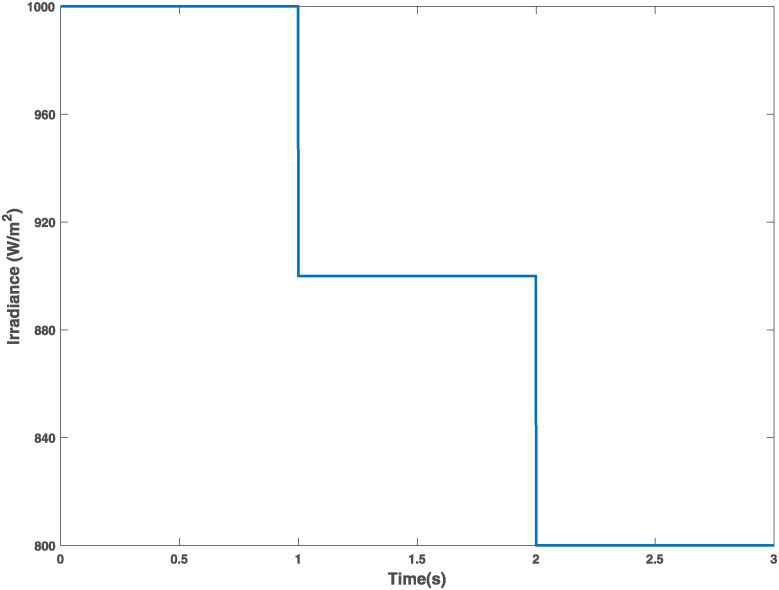
Case 2. Irradiance variations.

**Case 2 (Irradiance variations).** An initial solar irradiance of 1000 $\frac{W}{m^{2}}$ is considered, this value is maintained from 0 to 1 s; from 1 to 2 s, the irradiance decreases from 1000 $\frac{W}{m^{2}}$ to 900 $\frac{W}{m^{2}}$; in the interval from 2 to 3 s, the irradiance decreases from 900 $\frac{W}{m^{2}}$ to 800 $\frac{W}{m^{2}}$. A constant temperature of 25°C is considered. This scenario is shown in Fig 8.

**Fig 9:**
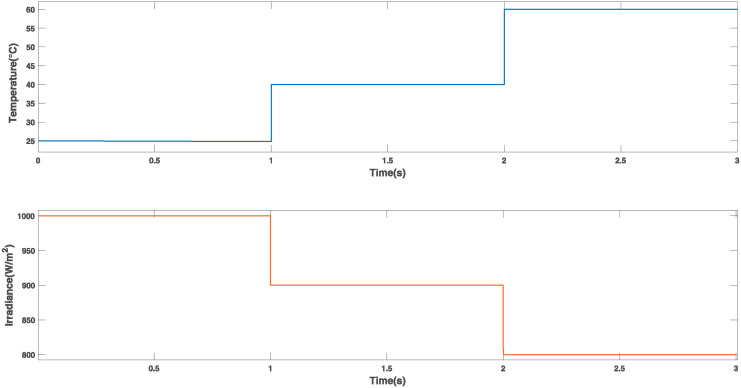
Case 3. Temperature and irradiance variations.

**Case 3 (Temperature and irradiance variations).** From 0 to 1 s, a temperature of 25°C and a solar irradiance of 1000 $\frac{W}{m^{2}}$ are considered; from 1 to 2 s, an increase of temperature from 25°C to 40°C is considered while the solar irradiance decreases from 1000 $\frac{W}{m^{2}}$ to 900 $\frac{W}{m^{2}}$; from 2 to 3 s, the temperature increases from 40°C to 60°C while the solar irradiance decreases from 900 $\frac{W}{m^{2}}$ to 800$\frac{W}{m^{2}}$. This scenario is illustrated in Fig 9.

The three considered scenarios are not realistic. In practice, such abrupt variations will never be seen. However, considering the above-mentioned cases allows us to challenge the controller, that is, if the controller is capable of operating satisfactorily in these situations, it will be effective when dealing with normal operating conditions (smooth variations of irradiance and temperature).

**Fig 10:**
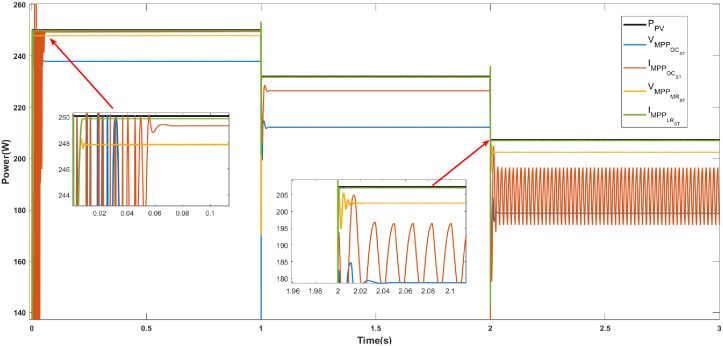
Case 1. Power generated considering a constant irradiance (1000*W*/*m*^2^) and a variable temperature using the reference signals $I_{MPP_{OC}}$, $V_{MPP_{OC}}$, $I_{MPP_{LR}}$, and $V_{MPP_{MR}}$.

**Fig 11:**
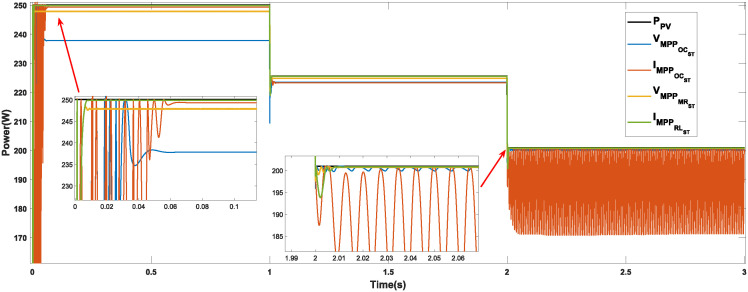
Case 2. Power generated considering a constant temperature (25°C) and a variable irradiance using the reference signals $I_{MPP_{OC}}$, $V_{MPP_{OC}}$, $I_{MPP_{LR}}$, and $V_{MPP_{MR}}$.

**Fig 12:**
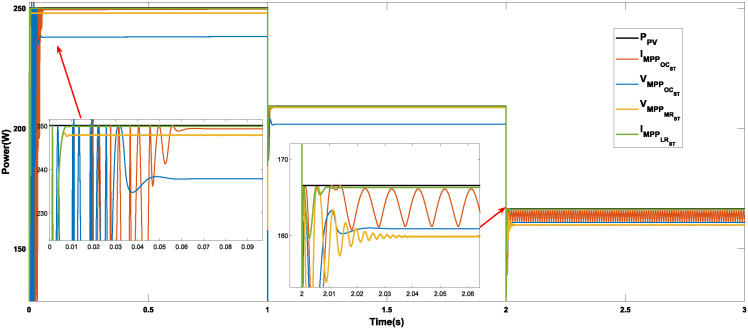
Case 3. Power generated considering variable irradiance and temperature using the reference signals $I_{MPP_{OC}}$, $V_{MPP_{OC}}$, $I_{MPP_{LR}}$, and $V_{MPP_{MR}}$.

Figs 10–12 show the power response of the system considering Cases 1, 2, and 3, respectively. In the figures, notation *P*_*PV*_ indicates the maximum power that the panel is capable of generating, while $V_{MPP_{OC_{ST}}}$, $I_{MPP_{OC_{ST}}}$, $V_{MPP_{MR_{ST}}}$ and $I_{MPP_{LR_{ST}}}$ denote the output power of the PV system using the ST-SMC considering the reference signals of voltage or current generated through the use of optimal operating parameters (OC), multiple regression (MR) or linear regression (LR).

To quantitatively assess the accuracy of the different reference signals and the performance of the ST-SMC strategy, we use the following error metrics:

- Integral of the Squared Error (ISE) $\int_{\tau_{ini}}^{\tau_{end}}{\left(e\left(t\right)\right)}^{2}dt$,
- Integral of Time multiplied by the Squared Error (ITSE) $\int_{\tau_{ini}}^{\tau_{end}}t{\left(e\left(t\right)\right)}^{2}dt$,
- Integral of Absolute magnitude of the Error (IAE) $\int_{\tau_{ini}}^{\tau_{end}}{\left|e(t)\right|}^{2}dt$,
- Integral of Time multiplied by Absolute Error (ITAE) $\int_{\tau_{ini}}^{\tau_{end}}t{\left|e(t)\right|}^{2}dt$.

For these metrics, the error *e*(*t*) is calculated as the difference between the MPP value and the delivered power; *τ*_*ini*_ and *τ*_*end*_, refer to the times at which the conditions of each case start and end.

The obtained results are described below.

**Case 1 (Temperature variations).** The response of the system under the ST-SMC for different reference signals under changing temperature conditions is shown in Fig 10. Note that the maximum power generated by the PV module in the interval from 0 to 1 s is 250.057 W. Note that, although the difference in response time for the different references is not significant, the system responds faster when considering the reference signal $I_{MPP_{LR}}$ (at 0.016 s approximately). Besides, the highest power was obtained also with this reference (250 W), while the lowest power was generated with the references $V_{MPP_{OC}}$ (237.8 W). With the temperature increase in the interval from 1 to 2 s, the maximum power generated by the PV system is 231.983 W. In this interval, the lowest power is generated with the reference $V_{MPP_{OC}}$ (178.71 W) while with the references $V_{MPP_{MR}}$ and $I_{MPP_{LR}}$, the generated power is very close to the maximum. Finally, when the temperature increases in the range of 2 to 3 s, the maximum power the photovoltaic system can generate is reduced to 207.928 W. In this scenario, oscillations can be observed in the signal generated with the reference $I_{MPP_{OC}}$, note also that with this reference the lowest power is generated. In this last interval, the highest power is generated when considering the reference $I_{MPP_{LR}}$ (207.067 W).

Table 3 shows the characteristics of the system response considering the operating conditions described in Case 1. Table 4 shows the numerical values of the error-based performance metrics corresponding to Case 1. Note that the best performance was obtained when considering the reference $I_{MPP_{LR}}$.

**Table 3:** Case 1. Characteristics of the system response in the intervals a) 0 to 1 s, b) 1 to 2 s, c) 2 to 3 s.

Reference	Response time (s)			Oscillation amplitude (W)			Maximum power (W)			Efficiency (%)		
	a	b	c	a	b	c	a	b	c	a	b	c
$V_{MPP_{OC}}$	0.06	0.04	0.04	0.03	0.02	0.05	237.865	178.71	160.87	94.01	96.34	96.55
$I_{MPP_{OC}}$	0.083	0.04	0.06	0.001	6.7	6.2	249.31	226.725	188.73	99.7	97.9	91.7
$V_{MPP_{MR}}$	0.015	0.012	0.026	0.092	0.017	3.2	247.91	231.227	202.532	99.3	99.90	97.72
$I_{MPP_{LR}}$	0.016	0.019	0.015	0.001	0.001	0.019	250.05	231.82	207.3	99.88	99.94	99.98

The theoretical maximum powers in each interval are: a) 250.057 W, b) 231.983 W, c) 207.928 W.

**Table 4:** Case 1. Error-based performance metrics for each algorithm.

Reference	ISE			ITSE			IAE			ITAE		
	a	b	c	a	b	c	a	b	c	a	b	c
$V_{MPP_{OC}}$	838.3	390.177	825.078	78.734	195.653	409.9	14.9607	19.677	28.614	6.136	9.89	14.223
$I_{MPP_{OC}}$	946.203	33.8696	428.44	13.3367	15.5826	209.903	5.2328	5.68	19.1838	0.3605	2.79	9.5448
$V_{MPP_{MR}}$	37.691	0.2545	22.539	1.126	0.022	11.0686	1.781	0.225	4.729	0.742	0.105	2.3515
$I_{MPP_{LR}}$	35.5167	0.067	0.07	0.0346	0.0082	0.0004	0.319	0.1446	0.033	0.0035	0.0636	0.0139

**Case 2 (Irradiance variations).** The response of the system under the ST-SMC for different reference signals under changing irradiance conditions is shown in Fig 11. As in the previous case, the maximum initial power that the system can generate is 250.057 W (in the interval from 0 to 1 s). The difference between the response times for the different reference signals is not significant. In all cases the system responds in less than 0.1 s. An important aspect to highlight is the fact that the power generated when considering the reference $I_{MPP_{LR}}$ is the closest to the maximum power in the range from 0 to 1 s (250 W), followed by $I_{MPP_{OC}}$ with 249.31 W, however, when the irradiance decreases to 800 W/*m*^2^ (interval from 2 to 3 s), the power generated with this reference not only decreases drastically, but also high frequency and magnitude oscillations can be observed. Note that, in this last interval, the difference between the power generated with the three remaining references is not significant. Table 5 shows the characteristics of the system response considering the operating conditions described in Case 2.

**Table 5:** Case 2. Characteristics of the system response in the intervals a) 0 to 1 s, b) 1 to 2 s, c) 2 to 3 s.

Reference	Response time (s)			Oscillation amplitude (W)			Maximum power (W)			Efficiency (%)		
	a	b	c	a	b	c	a	b	c	a	b	c
$V_{MPP_{OC}}$	0.06	0.04	0.04	0.05	0.001	0.93	237.865	223.48	200.8	94.01	99.04	99.75
$I_{MPP_{OC}}$	0.083	0.05	0.04	0.001	0.01	3.625	249.31	222.96	188.787	99.7	98.8	93.9
$V_{MPP_{MR}}$	0.04	0.02	0.012	0.092	0.050	0.015	248.596	224.99	200.89	99.29	99.73	99.96
$I_{MPP_{LR}}$	0.016	0.02	0.024	0.001	0.005	0.001	250.05	225.6	200.8	99.88	99.99	99.91

The theoretical maximum powers in each interval are: a) 250.057 W, b) 225.608 W, c) 200.977 W.

Table 6 shows the numerical values of the error-based performance metrics corresponding to Case 2. Note that, in general, the best performance was obtained when the references $V_{MPP_{MR}}$ and $I_{MPP_{LR}}$ are considered.

**Table 6:** Case 2. Error-based performance metrics for each algorithm.

Reference	ISE			ITSE			IAE			ITAE		
	a	b	c	a	b	c	a	b	c	a	b	c
$V_{MPP_{OC}}$	838.3	4.663	0.35	81.40	2.27	0.171	14.9607	2.147	0.5	6.136	1.066	0.248
$I_{MPP_{OC}}$	946.203	5.39093	502.963	13.3367	2.71531	260.45	5.2328	2.3151	16.3399	0.3605	1.1651	8.3174
$V_{MPP_{MR}}$	37.691	0.384	0.012	1.126	0.192	0.002	1.781	0.619	0.072	0.742	0.31	0.034
$I_{MPP_{LR}}$	35.5167	0.165	0.5	0.0346	0.0005	0.009	0.319	0.04	0.18	0.0035	0.0005	0.061

**Case 3 (Temperature and irradiance variations).** The system response under the ST-SMC for different reference signals under variations of irradiance and temperature is shown in Fig 12. Note that a poor performance in terms of the generated power is obtained when the reference $V_{MPP_{OC}}$ is considered. Also, as in the previous cases, in the interval from 2 to 3s, an oscillatory response can be observed when the reference $I_{MPP_{OC}}$ is considered. Table 7 shows the characteristics of the system response considering the operating conditions of Case 3.

**Table 7:** Case 3. Characteristics of the system response in the intervals a) 0 to 1 s, b) 1 to 2 s, c) 2 to 3 s.

Reference	Response time (s)			Oscillation amplitude (W)			Maximum power (W)			Efficiency (%)		
	a	b	c	a	b	c	a	b	c	a	b	c
$V_{MPP_{OC}}$	0.06	0.04	0.04	0.05	0.001	0.05	237.865	201.71	160.87	94.01	96.34	96.55
$I_{MPP_{OC}}$	0.08	0.06	0.04	0.001	0.001	0.81	249.31	209.2	164.599	99.7	99.3	98.5
$V_{MPP_{MR}}$	0.04	0.02	0.07	0.034	0.082	0.015	248.596	208.797	159.977	99.29	99.75	96.01
$I_{MPP_{LR}}$	0.014	0.014	0.012	0.006	0.014	0.018	249.86	209.109	166.28	99.81	99.96	99.91

The theoretical maximum powers in each interval are: a) 250.057 W, b) 209.296 W, c) 166.541 W.

Table 8 shows the numerical values of the error-based performance metrics corresponding to Case 3. Note that, in general, the reference that allows obtaining the best performance is $I_{MPP_{LR}}$.

**Table 8:** Case 3. Error-based performance metrics for each algorithm.

Reference	ISE			ITSE			IAE			ITAE		
	a	b	c	a	b	c	a	b	c	a	b	c
$V_{MPP_{OC}}$	838.3	59.567	34.162	78.34	29.023	16.089	14.7648	7.663	5.739	6.0103	3.81	2.837
$I_{MPP_{OC}}$	946.203	1.307	4.9528	13.3367	0.0071	1.4486	5.2328	0.1189	1.4747	0.3605	0.01259	0.6858
$V_{MPP_{MR}}$	37.691	0.472	45.387	1.126	0.123	21.57	1.781	0.519	6.632	0.742	0.248	3.2839
$I_{MPP_{LR}}$	35.5167	0.448	0.315	0.0346	0.0019	0.0079	0.319	0.079	0.153	0.0035	0.017	0.06

As we have observed, the ST-SMC considering the four reference signals allows solving the MPPT problem, being the reference $I_{MPP_{LR}}$ with which, in general, the best performance is achieved.

In order to assess the effectiveness of the proposal with respect to other MPPT strategies, the next section presents a comparative study considering the classical methods P&O and INC as well as two recently introduced ANN-PID algorithms. The comparison will be developed considering the ST-SMC with the references that allowed better performance: $I_{MPP_{LR}}$ and $V_{MPP_{MR}}$.

## Comparison with P&O, INC and ANN-PID algorithms

This section presents a comparative analysis of the performance of the ST-SMC considering the reference signals $I_{MPP_{LR}}$ and $V_{MPP_{MR}}$ obtained from the regression models with respect to the classical P&O and INC methods. In addition, to evaluate the performance of the proposed control strategy with respect to relevant and recent state-of-the-art solutions, a comparative analysis considering the hybrid techniques studied in [27, 28] is presented.

In [27], Levenberg-Marquardt (LM), Bayesian Regularization (BR), and Scaled Conjugate Gradient (SCG) training algorithms for ANNs are used to estimate a voltage reference to solve the problem of MPPT of a PV system; a comparative analysis of the performance of the three algorithms is presented. The paper concludes that the ANN training with the LM algorithm performs better than the BR and SCG training algorithms in overall data processing. In [28], an MPPT method based on ANN using BR training to generate the PV current reference for the MPPT problem is proposed. In broad outline, both ANNs described in [27, 28] are used to generate a dataset from a Simulink MATLAB diagram of the PV system, and by analyzing the output characteristics of a solar cell, a neural network-based algorithm allows the tracking of the MPP.

The hybrid ANN-based algorithms described above are considered to develop the comparison. Both hybrid algorithms are constituted of an ANN that generates references either of voltage (using 10 neurons in its hidden layer) [27] or current (using 15 neurons in its hidden layer) [28]. Based on the generated references, a PID controller is used to solve the MPPT problem. Both ANNs use the PV irradiance and temperature as independent variables to estimate their output variable.

It is important to point out that the considered hybrid methods were chosen to develop the comparative analysis since they constitute novel methods that use intelligent MPPT algorithms and are based on the well studied ANN, see for instance [27–31].

**Fig 13:**
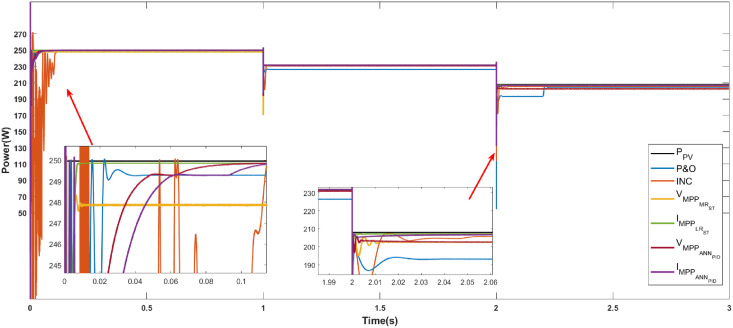
Case 1. Power generated considering a constant irradiance (1000 *W*/*m*^2^) and a variable temperature through the MPPT algorithms P&O, INC, ST-SMC and ANN-PID.

**Fig 14:**
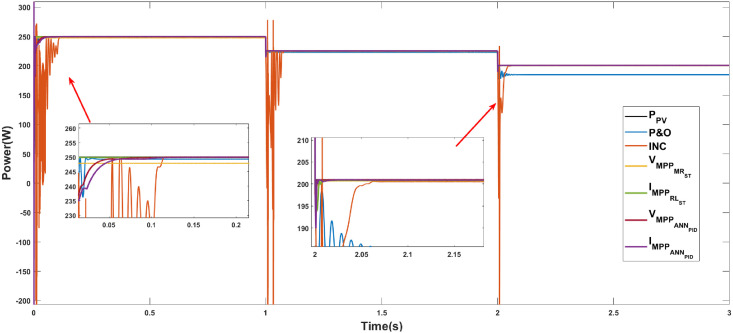
Case 2. Power generated considering a constant temperature (25°C) and a variable irradiance through the MPPT algorithms P&O, INC, ST-SMC and ANN-PID.

**Fig 15:**
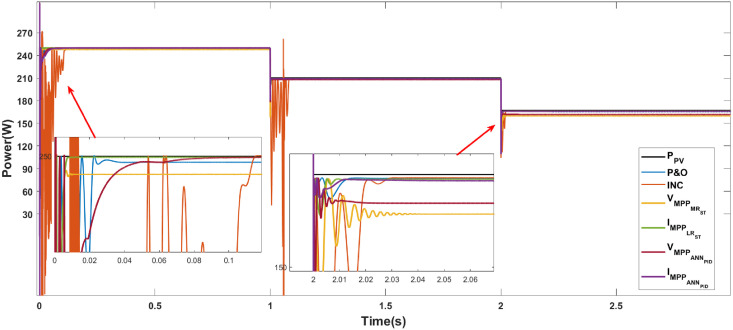
Case 3. Power generated considering variable conditions of irradiance and temperature through the MPPT algorithms P&O, INC, ST-SMC and ANN-PID.

The three cases described in the previous section are considered for the development of the comparative analysis. Figs 13–15 show the simulation outcomes corresponding to Case 1, Case 2, and Case 3, respectively. In these figures, notation $V_{MPP_{ANN_{PID}}}$ refers to the system response generated through the LM algorithm proposed in [27] which generates a voltage reference signal, and $I_{MPP_{ANN_{PID}}}$ stands for the system response under the algorithm proposed in [28] which generates a current reference signal.

**Case 1 (Temperature variations).** The system response considering the algorithms P&O, INC, ST-SMC and ANN-PID under changing temperature conditions is shown in Fig 13. Table 9 shows the numerical values of the response characteristics. Note that a power close to the desired one is generated when the algorithms ST-SMC (with reference $I_{MPP_{LR}}$) and ANN-PID are used, the former being the one that responds faster in the first interval. In 2 of the 3 intervals, the poorest performance in terms of generated power is obtained with the P&O algorithm. Although INC algorithm generates higher power compared to the P&O method, it generates an undesirable oscillatory transient response in each interval.

**Table 9:** Case 1. Comparison of the algorithms P&O, INC, ST-SMC, and ANN-PID.

Algorithm	Response time (s)			Oscillation amplitude (W)			Maximum power (W)			Efficiency (%)		
	a	b	c	a	b	c	a	b	c	a	b	c
*P*&*O*	0.07	0.06	0.25	0.01	0.01	0.01	249.28	226.4	204.6	99.2	97.58	97.55
INC	0.12	0.05	0.1	124.05	31.978	35.5	250.05	230.92	205.57	96.54	99.45	99.01
$V_{MPP_{MR}}$	0.015	0.012	0.026	0.092	0.017	3.2	247.91	231.227	202.532	99.3	99.90	97.72
$I_{MPP_{LR}}$	0.016	0.019	0.015	0.001	0.001	0.019	250.05	231.82	207.3	99.88	99.94	99.98
$V_{MPP_{ANN_{PID}}}$	0.13	0.1	0.08	0.015	0.041	0.077	249.86	230.86	202.58	99.65	99.51	97.7
$I_{MPP_{ANN_{PID}}}$	0.11	0.005	0.014	0.003	0.003	0.004	249.84	231.35	206.043	99.95	99.9	99.09

Characteristics of the system response in the intervals a) 0 to 1 s, b) 1 to 2 s, c) 2 to 3 s. The theoretical maximum powers in each interval are: a) 250.057 W, b) 231.983 W, c) 207.928 W.

Table 10 shows the error-based performance metrics corresponding to Case 1. As we have seen, the worst-performing algorithms are P&O, INC, and $V_{MPP_{MR}}$ while no significant differences can be observed among the remaining ones.

**Table 10:** Case 1. Error-based performance metrics for each algorithm.

Algorithm	ISE			ITSE			IAE			ITAE		
	a	b	c	a	b	c	a	b	c	a	b	c
*P*&*O*	163	31.4	48.109	1.36	15.57	7.65	1.975	5.58	5.07	0.395	2.77	1.59
*INC*	1648	5.85	9.35	45.413	0.61	0.97	8.68	1.267	1.62	0.317	0.54	0.523
$V_{MPP_{MR}}$	37.691	0.2545	22.539	1.126	0.022	11.0686	1.781	0.225	4.729	0.742	0.105	2.3515
$I_{MPP_{LR}}$	35.5167	0.067	0.07	0.0346	0.0082	0.0004	0.319	0.1446	0.033	0.0035	0.0636	0.0139
$V_{MPP_{ANN_{PID}}}$	41.6065	1.394	22.95	0.1372	0.6433	11.352	0.8644	1.138	4.764	0.1	0.5671	2.382
$I_{MPP_{ANN_{PID}}}$	63.099	0.09127	0.7176	0.1417	0.0359	0.3463	0.8362	0.2692	0.8148	0.03329	0.134	0.4160

**Case 2 (Irradiance variations).** The system response under the algorithms P&O, INC, ST-SMC and ANN-PID under changing irradiance conditions is shown in Fig 14. Table 11 shows the numerical values of the response characteristics. Note that the characteristics of the generated power signal are similar to those of the previous case, that is, a transient oscillatory response with large magnitude overshoots is observed when considering the INC method. In 2 of the 3 intervals, the worst performance in terms of generated power is obtained when considering the P&O algorithm. As in the previous case, a satisfactory performance is achieved when the algorithms ST-SMC (with reference $I_{MPP_{LR}}$) and ANN-PID (for both current and voltage references) are considered. Note also that with the ST-SMC, considering the voltage reference $V_{MPP_{LR}}$, although a slightly lower power is generated, good performance is observed in general. Table 12 shows the error-based performance metrics corresponding to Case 2.

**Table 11:** Case 2. Comparison of the algorithms P&O, INC, ST-SMC, and ANN-PID.

Algorithm	Response time (s)			Oscillation amplitude (W)			Maximum power (W)			Efficiency (%)		
	a	b	c	a	b	c	a	b	c	a	b	c
*P*&*O*	0.07	0.05	0.19	0.01	0.01	0.02	249.28	223.25	185.06	99.2	98.95	92.11
INC	0.12	0.1	0.08	124.05	321.32	80.28	250.05	225.46	200.5	96.54	97.29	98.5
$V_{MPP_{MR}}$	0.04	0.02	0.012	0.092	0.050	0.015	248.596	224.99	200.89	99.29	99.73	99.96
$I_{MPP_{LR}}$	0.016	0.02	0.024	0.001	0.005	0.001	250.05	225.6	200.8	99.88	99.99	99.91
$V_{MPP_{ANN_{PID}}}$	0.1	0.003	0.003	0.003	0.002	0.002	249.82	225.647	200.938	99.65	99.65	99.65
$I_{MPP_{ANN_{PID}}}$	0.11	0.02	0.014	0.003	0.002	0.0001	249.84	225.62	200.778	99.95	99.98	99.99

Characteristics of the system response in the intervals a) 0 to 1 s, b) 1 to 2 s, c) 2 to 3 s. The theoretical maximum powers in each interval are: a) 250.057 W, b) 225.608 W, c) 200.977 W.

**Table 12:** Case 2. Error-based performance metrics for each algorithm.

Algorithm	ISE			ITSE			IAE			ITAE		
	a	b	c	a	b	c	a	b	c	a	b	c
*P*&*O*	163	5.57	252.48	1.36	2.785	126.34	1.975	2.35	15.85	0.395	1.18	7.95
*INC*	1648	1126.21	9.35	45.413	24.16	3.178	8.68	6.13	2.99	0.317	0.228	0.268
$V_{MPP_{MR}}$	37.691	0.384	0.012	1.126	0.192	0.002	1.781	0.619	0.072	0.742	0.31	0.034
$I_{MPP_{LR}}$	35.5167	0.165	0.5	0.0346	0.0005	0.009	0.319	0.04	0.18	0.0035	0.0005	0.061
$V_{MPP_{ANN_{PID}}}$	62.7832	0.00699	0.1192	0.1291	0.00023	0.0559	0.8392	0.02522	0.3360	0.0845	0.0106	0.1672
$I_{MPP_{ANN_{PID}}}$	63.099	0.0887	0.1618	0.1417	0.00018	0.03125	0.8362	0.02764	0.2627	0.03329	0.00697	0.1248

**Case 3 (Temperature and irradiance variations).** The system response under the algorithms P&O, INC, ST-SMC and ANN-PID under changing temperature and irradiance conditions is shown in Fig 15. Table 13 shows the numerical values of the response characteristics. This case represents the most challenging of the considered scenarios, especially in the range of 2 to 3 s, where the highest temperature (60°C) and the lowest irradiance (800 $\frac{W}{m^{2}}$) are considered. In the intervals from 0 to 1 s and from 1 to 2 s, there is no significant difference between the values of generated power with the considered methods. However, in the 2 to 3 s interval, the lowest power values are generated when considering voltage references($V_{MPP_{MR_{ST}}}$, $V_{MPP_{ANN_{PID}}}$) while current references ($I_{MPP_{LR_{ST}}}$, $I_{MPP_{ANN_{PID}}}$) reach the MPTT without significant differences with the classical algorithms. Table 14 shows the error-based performance metrics corresponding to Case 3. Note that the highest error values are obtained for the INC algorithm.

**Table 13:** Case 3. Comparison of the algorithms P&O, INC, ST-SMC, and ANN-PID.

Algorithm	Response time (s)			Oscillation amplitude (W)			Maximum power (W)			Efficiency (%)		
	a	b	c	a	b	c	a	b	c	a	b	c
*P*&*O*	0.07	0.05	0.04	0.01	0.01	0.81	249.28	209.2	166.25	99.2	99.94	99.73
INC	0.12	0.12	0.08	124.05	167.716	51.71	250.05	208.82	166.5	96.54	97.76	99.75
$V_{MPP_{MR}}$	0.04	0.02	0.07	0.034	0.082	0.015	248.596	208.797	159.977	99.29	99.75	96.01
$I_{MPP_{LR}}$	0.014	0.014	0.012	0.006	0.014	0.018	249.86	209.109	166.28	99.81	99.96	99.91
$V_{MPP_{ANN_{PID}}}$	0.1	0.007	0.018	0.003	0.031	0.013	249.82	207.934	161.94	99.65	99.36	97.12
$I_{MPP_{ANN_{PID}}}$	0.11	0.018	0.012	0.003	0.002	0.014	249.84	209.208	166.238	99.65	99.89	99.66

Characteristics of the system response in the intervals a) 0 to 1 s, b) 1 to 2 s, c) 2 to 3 s. The theoretical maximum powers in each interval are: a) 250.057 W, b) 209.296 W, c) 166.541 W.

**Table 14:** Case 3. Error-based performance metrics for each algorithm.

Algorithm	ISE			ITSE			IAE			ITAE		
	a	b	c	a	b	c	a	b	c	a	b	c
*P*&*O*	163	0.104	0.58	1.36	0.004	0.272	1.975	0.107	0.444	0.395	0.044	0.243
*INC*	1648	602.58	13.388	45.413	26.45	0.088	8.68	4.69	0.41	0.317	0.392	0.0057
$V_{MPP_{MR}}$	37.691	0.472	45.387	1.126	0.123	21.57	1.781	0.519	6.632	0.742	0.248	3.2839
$I_{MPP_{LR}}$	35.5167	0.448	0.315	0.0346	0.0019	0.0079	0.319	0.079	0.153	0.0035	0.017	0.06
$V_{MPP_{ANN_{PID}}}$	62.7832	1.677	11.20	0.1291	0.8007	5.561	0.8392	1.2627	3.3292	0.0845	0.0106	1.6682
$I_{MPP_{ANN_{PID}}}$	63.099	0.067	0.8596	0.1417	0.0171	0.4099	0.8362	0.1769	0.9113	0.03329	0.00697	0.4527

## Discussion

In the comparative analysis, different methods to solve the MPPT problem were evaluated. Two classical algorithms: P&O and INC, and two hybrid methods: ST-SMC with references generated through linear and multiple regressions, and PID with references generated through ANN. The algorithms were challenged to operate under abrupt variations of irradiance and temperature. As a result, the classical methods were outperformed by the hybrid approaches. Oscillatory responses with large overshoots were obtained with the INC algorithm, resulting in longer settling times, while the lowest values of generated power were obtained with the P&O method.

Both classical algorithms work by dynamically adjusting the PV system operation to reach the MPP under changing conditions. The P&O algorithm is a simpler and more widely implemented approach that relies on disturbance and power observations to adjust its operation, while the INC algorithm uses more detailed information to calculate the slope of the power-voltage (*P*_*pv*_-*V*_*pv*_) curve with the objective of finding a maximum point, which makes its operation more complex compared to the P&O method.

The analyzed hybrid MPPT methods were shown to be more efficient in terms of generated power and response time. An important aspect of their operation is determining the reference signals that a controller must allow to track. For the ST-SMC proposed in this work, it was shown that linear and multiple regressions are efficient in determining them.

The computational cost of an algorithm can be determined by the required number of mathematical operations between scalars [58]. The multiple regression to determine the voltage reference $V_{MPP_{MR}}$ in terms of irradiance and temperature involves two multiplications and two additions. The linear regression to determine the current reference $I_{MPP_{LR}}$ in terms of irradiance involves only one multiplication and one addition.

On the other side, for the hybrid method that uses an ANN to generate a voltage reference, the following operations are required:

1. Normalization of two input variables in the interval [-1,1], which requires six subtractions, two multiplications, two divisions, and one addition.
2. Computation of the ANN output from two input neurons, ten hidden layer neurons, and one output neuron; this involves 20 multiplications and 11 additions. The resulting scalar is the argument of the hyperbolic tangent activation nonlinear function, which is the ANN output.
3. Calculation of the denormalizing process for the output variable (mapping from the interval [-1,1] to the original one), which involves 3 subtractions, one multiplication, one division, and one addition.

The whole process involves then 9 subtractions, 23 multiplications, three divisions, 13 additions, and one nonlinear operation. In addition, 30 parameters of the ANN and 12 parameters for normalizing the input data and denormalizing the output data need to be stored in memory.

Similarly, to generate the current reference for the ANN-PID method, 9 subtractions, 33 multiplications, two divisions, 18 additions, and one nonlinear operation are required. In this case, 45 parameters of the ANN and 12 parameters for normalizing the input data and denormalizing the output data need to be stored in memory.

It is obvious that the proposed approach to generate the reference signals through multiple and linear regression is much simpler in computational terms than the method that uses ANN. However, some comments regarding the control approaches used to track these reference signals are in order.

An important aspect that must be considered when choosing a control strategy is the trade-off between computational cost and performance. On one hand, it is well known that the ST-SMC proposed in this work to solve the MPPT problem offers robustness and disturbance rejection, however, its higher computational demand may limit its applicability in resource-constrained embedded systems [59]. On the other hand, a PID controller provides a balance between performance and computational complexity, making it more suitable for some real-time embedded applications [60]. However, among its drawbacks we can mention the tuning complexity and limited robustness against disturbances and external noise.

The proposal of combing the low-complexity regression method with the powerful ST-SMC constitutes a resourceful approach to solve the MPPT problem that can be implemented in resource-constrained embedded systems.

From Tables 9, 11 and 13, we can observe that the algorithms that are least efficient in extracting the MPP are INC and P&O, followed by the hybrid algorithms based on voltage references ($V_{MPP_{ANN_{PID}}}$ and $V_{MPP_{LR_{ST}}}$). The most efficient algorithms are those hybrid based on current references; in most of the intervals, the efficiency obtained by $I_{MPP_{LR_{ST}}}$ is better than $I_{MPP_{ANN_{PID}}}$, which suggests that the current reference $I_{MPP_{LR}}$ determined by linear regression, although not better (RMSE = 0.0218) than that obtained by the ANN (RMSE = 0.0061), is counterbalanced by a better controller to reach the MPP.

In general, the ST-SMC algorithm with the current reference $I_{MPP_{LR}}$ has shown to have satisfactory performance, surpassing the other analyzed MPPT techniques in most cases. It should be mentioned that the superiority of the proposed method (in terms of efficiency, oscillations and response time) with respect to the ANN-based methods is not substantial. However, the complexity involved in the generation of the reference signal is lower compared to the one of the ANN-based methods. Besides, the ST-SMC algorithm with the current reference $I_{MPP_{LR}}$ does not require the implementation of a temperature sensor, which reduces operating costs.

To implement the proposed ST-SMC algorithm in a real PV system, an irradiance sensor, a Boost converter circuit and measurements of *I*_*pv*_ and *V*_*pv*_ are required. The control signal can be generated using a PC equipped with a data acquisition card (see for instance [52, 54, 61]), or alternatively, using an embedded system with microcontrollers (see for example [14, 15]).

## Conclusions

A novel strategy for solving the MPPT problem via the ST-SMC is presented. The novelty of the method relies on the proposed approaches to define the reference signals that the ST-SMC requires for proper tracking of the MPP in a PV system under abrupt variations of irradiance and temperature. The effectiveness of the proposed approach is verified through numerical simulations carried out in MATLAB. For the sake of completeness, a comparative analysis with the methods P&O, INC and ANN-PID is presented. From the simulations, it has been observed that, in general, the best approach to track the MPP, in view of the response time and efficiency, is the ST-SMC with the current reference generated through a linear regression model. In fact, with this proposal, no overshoots nor oscillations of important magnitude are observed in the system response.

A major challenge of hybrid algorithms such as the one proposed in this work that uses irradiance measurements is the difficulty of solving the MPPT problem under partial shading conditions. The difficulty lies in the impossibility of knowing exactly the shaded areas within the panel and their corresponding irradiance values. To solve this problem, the implementation of hybrid methods based on metaheuristics is proposed as a future work.

## Supporting information

S1 Appendix Stability analysis [62–65]. (ZIP)

S1 File MATLAB files. (ZIP)
