# Supplementary material for: A novel strategy for the MPPT in a photovoltaic system via sliding modes control
Source: PLoS One. 2024 Dec 13;19(12):e0311831. doi: 10.1371/journal.pone.0311831 (PMC11642983; doi:10.1371/journal.pone.0311831)
Supplement: S1 Appendix — (ZIP) [file pone.0311831.s001.zip › Appendix.pdf]

S1 Appendix: Stability analysis

This appendix presents the proof of convergence of the ST-SMC algorithm for both sliding surfaces s_1 and s_2.

For the sliding surface s_1 defined in (5), it must be shown that by using the controller u defined in (4), (7)-(8), it is guaranteed that I_{pv} converges to I_{MPP} in finite time. Taking the time-derivative of s_1 one has:

$$\frac{ds_1}{dt} = \frac{I_{pv}}{dt} - \frac{dI_{MPP}}{dt}$$

By substituting the system dynamics (2) and the controller u defined in (4), (7)-(8) one gets:

$$\frac{ds_1}{dt} = \frac{V_{pv}}{L} - \frac{V_s}{L}\left(\frac{V_{pv}}{V_s} + \lambda|s_1|^{\frac{1}{2}} sign(s_1) + \Upsilon\int_0^t sign(s_1(d\tau))d\tau\right) - \frac{dI_{MPP}}{dt}$$

simplifying,

$$\frac{ds_1}{dt} = -\frac{V_s}{L}\left(\lambda|s_1|^{\frac{1}{2}} sign(s_1) + \Upsilon\int_0^t sign(s_1(d\tau))d\tau\right) - \frac{dI_{MPP}}{dt}$$

By defining the variables $x_1 = s_1$, $x_2 = \int_0^t sign(s_1(d\tau))d\tau$ and $\varrho = -\frac{dI_{MPP}}{dt}$, the above equation can be written as:

$$\begin{aligned} \dot{x}_1 &= -k_1|x_1|^{\frac{1}{2}} sign(x_1) + x_2 + \varrho \\ \dot{x}_2 &= -k_2 sign(x_1) \end{aligned} \tag{A. I}$$

where $k_1 = \frac{V_s\lambda}{L}$ and $k_2 = \frac{V_s\Upsilon}{L}$.

Exhaustive research has been conducted to prove the stability of the super twisting algorithm described by (A. I), see for instance [62]-[65] Following [62], the finite-time convergence of the super twisting algorithm without perturbations, i.e, $\varrho = 0$, can be proven by choosing the strict Lyapunov function:

$$V(x) = \zeta^T P\zeta,$$

where $\zeta^T = [\zeta_1,\ \zeta_2] = \left[|x_1|^{\frac{1}{2}} sign(x_1),\ x_2\right]$ and P is a constant, symmetric and positive definite matrix. Taking the time-derivative of $V(x)$ along the trajectories of the unperturbed system (A. I) one gets $\dot{V}(x) = \zeta^T P\dot{\zeta} + \dot{\zeta}^T P\zeta$, where:

$$\dot{\zeta} = \frac{1}{|\zeta_1|}A\zeta, \quad A = \begin{bmatrix} -\frac{1}{2}k_1 & \frac{1}{2} \\ -k_2 & 0 \end{bmatrix}$$

Then, the time-derivative of the Lyapunov function is given by:

$$\dot{V} = -|x_1|^{\frac{1}{2}}\zeta^T Q\zeta$$

where P and Q are related by the Algebraic Lyapunov Equation (ALE) $A^T P + PA = -Q$. Since A is Hurwitz if and only if $k_1 > 0$ and $k_2 > 0$, for every $Q = Q^T > 0$ there exists a unique solution $P = P^T$ of the ALE. This result shows that the stability of the equilibrium $x = 0$ of (A. I) is completely determined by the stability of the matrix A, i.e., the equilibrium is stable if and only if the eigenvalues of A have negative real part. This condition is satisfied for $k_1 > 0$, $k_2 > 0$. Moreover, a trajectory starting at x_0 at time $t = 0$ reaches the origin in a time smaller than $T(x_0)$ given by:

$$T(x_0) = \frac{2}{\sigma}V^{\frac{1}{2}}(x_0), \quad \sigma = \frac{\lambda_{\min}^{\frac{1}{2}}(P)\lambda_{\min}(Q)}{\lambda_{\max}(Q)}$$

In the case under study, the stability condition $k_1 = \frac{V_s \lambda}{L} > 0$ and $k_2 = \frac{V_s \Upsilon}{L} > 0$ is satisfied. In view of (5), the stability of the equilibrium $x = 0$ implies the finite time convergence of I_{pv} to I_{MPP}. Note that the derived stability analysis was developed for the perturbation-free system, i.e., $\varrho = -\frac{dI_{MPP}}{dt} = 0$, which, for the cases analyzed in this work is always satisfied except for the instants at which the irradiance and/or temperature changes. For the case $\varrho = -\frac{dI_{MPP}}{dt} \neq 0$, additional restrictions on the controller gains λ and Υ must be imposed, see for instance [62] and [65].

Now, similarly, for the sliding surface s_2 defined in (6), it must be shown that by using the controller u defined in (4), (7)-(8), it is guaranteed that V_{pv} converges to V_{MPP} in finite time. Taking the time-derivative of s_2 one has:

$$\frac{ds_2}{dt} = \frac{V_{pv}}{dt} - \frac{dV_{MPP}}{dt}$$

By using the relation $V_{pv} = RI_{pv}$ one gets

$$\frac{ds_2}{dt} = R\frac{I_{pv}}{dt} - \frac{dV_{MPP}}{dt}$$

Then, by substituting the system dynamics (2) and the controller u defined in (4), (7)-(8) one gets again the system (A. I) but now the involved variables are $x_1 = s_2$, $x_2 = \int_0^t sign(s_2(d\tau))d\tau$ and $\varrho = -\frac{dV_{MPP}}{dt}$. In this case, $k_1 = \frac{RV_s\lambda}{L}$ and $k_2 = \frac{RV_s\Upsilon}{L}$. The stability condition $k_1 > 0$, $k_2 > 0$ is also satisfied and the proof follows the same lines as before. Then, the finite time convergence of V_{pv} to V_{MPP} is guaranteed.
