# Supplementary material for: A novel strategy for the MPPT in a photovoltaic system via sliding modes control
Source: PLoS One. 2024 Dec 13;19(12):e0311831. doi: 10.1371/journal.pone.0311831 (PMC11642983; doi:10.1371/journal.pone.0311831)
Supplement: S1 File — (ZIP) [file pone.0311831.s002.zip › S2 MATLAB files.pdf]

S2 MATLAB files

The MATLAB files required to reproduce the results presented in this paper can be downloaded from: https://doi.org/10.5281/zenodo.11372189
